# Consumer Preferences for Cheese Products with Quality Labels: The Case of Parmigiano Reggiano and Comté

**DOI:** 10.3390/ani12101299

**Published:** 2022-05-18

**Authors:** Davide Menozzi, Ching-Hua Yeh, Elena Cozzi, Filippo Arfini

**Affiliations:** 1 Department of Food and Drug, University of Parma, Via J. F. Kennedy 6, 43125 Parma, Italy; davide.menozzi@unipr.it (D.M.); elena.cozzi@unipr.it (E.C.); 2 Institute for Food and Resource Economics, University of Bonn, Nußallee 21, 53115 Bonn, Germany; 3 Department of Economics and Management, University of Parma, Via J. F. Kennedy 6, 43125 Parma, Italy; filippo.arfini@unipr.it

**Keywords:** willingness to pay, protected-designation-of-origin (PDO) cheese, discrete choice experiment, Mountain Product, organic label

## Abstract

**Simple Summary:**

A number of food quality schemes may be associated with dairy products, promoting specific production methods (e.g., organic) as well as products obtained from a narrow area of origin (e.g., protected designation of origin, PDO). The coexistence of multiple labels is less investigated in the literature, even if its role in consumer studies could reveal interesting hints for both the stakeholders in the production chains (e.g., to target marketing strategies) and for consumers, who could access more precise information about the origin and the production processes. In this study, we provide evidence of consumers’ preferences and willingness to pay for cheese products (Comté and Parmigiano Reggiano) in two EU countries (France and Italy, respectively). We found how consumers’ choices varied by combining the PDO label with other quality features (i.e., the organic label in France, and the Mountain Product label in Italy). Still, price remained the most important factor influencing consumers’ decisions; however, we found how combined food quality labels could influence consumers’ choices. We found different market segments in the two countries presenting more positive attitudes towards quality-labeled food products, thus providing stakeholders with helpful information on how to develop tailored marketing strategies.

**Abstract:**

The paper examined the potential demand for a food specialty dairy product, cheese, with alternative multiple labels. A random-parameter logit model was applied to interpret the results of online discrete choice experiments (DCE) for the elicitation of the preference of the cheese consumers surveyed in two European countries, France (*n* = 400) and Italy (*n* = 408). We analyzed consumers’ choices of quality-labeled cheeses, i.e., protected-designation-of-origin (PDO)-labeled Parmigiano Reggiano and Comté. Other features were tested, such as organic (Comté) and Mountain Product (Parmigiano Reggiano) labels, companies’ brands and price. The paper contributes to the literature on credence attributes by examining consumers’ willingness to pay (WTP) for differentiated cheese products in two EU countries, and by identifying the effects of personal characteristics, in terms of socio-demographics and level of product involvement, on the differences in preferences. The results show that price was the most important attribute in both countries, followed by the PDO quality label, particularly when paired with the second quality feature. Two cheese consumer segments were identified via latent class models in each country, helping producers to improve their marketing of agri-food products with a high gastronomic value and differentiation potential.

## 1. Introduction

The European Union (EU) is pursuing the protection of geographical indications (GIs) with the objective of promoting the uniqueness of agricultural products and foodstuffs strictly linked to their geographical origin, the traditional know-how and processing procedures. Since the 1992 Council Regulations (EEC No. 2081/92 and No. 2082/92), the European food market has been enriched with new quality schemes. Besides the well-known protected designation of origin (PDO), protected geographical indication (PGI) and traditional specialty guaranteed (TSG), other schemes have been introduced, such as the optional quality term Mountain Product (EU Parliament and Council Regulation No. 1151/2012, art. 31, and further specified with Regulation No. 665/2014). Indeed, EU Regulation No. 1151/2012 reserves the use of the term Mountain Product for food products produced and processed in mountain areas [1]. Along with the abovementioned EU quality schemes, voluntary certifications at the international and national levels also exist, providing consumers with extra information about the product’s quality features. The EU organic certification (introduced with Council Regulation EEC No. 2092/91 and currently under EU Regulation 2018/848 of the European Parliament and of the Council), and the national organic labels available in some EU countries (e.g., Agriculture Biologique in France) can be adopted by producers to make explicit their efforts in minimizing environmental impacts and increasing animal welfare.

In general, food quality schemes (FQSs) represent not only a clear advantage in terms of price premiums for producers [2] or added value, generating positive socio-economic impacts for the territories where the products stem from [3,4,5,6], but encompass a large variety of ecosystem services too [7]. In this light, recent projects have stressed the great potential of FQSs in terms of sustainability when compared with corresponding reference products [8]. This feature is notably important and should be stressed and promoted in order to pursue production patterns which are more respectful of the planetary boundaries, of local biodiversity and traditional know-how.

Along with production and processing methods, consumption patterns also have to be re-thought to take into greater consideration the planetary boundaries and the current emergencies faced by the food sector/chains. In this context, FQSs may benefit consumers from different perspectives: they assure and communicate high-quality standards, as defined and fixed by the certification process [9,10] and allow consumer to make an informed choice, thereby contributing to fair competition for producers and providing reliable information and lower transaction costs [11,12].

Several studies have been conducted to explore and reveal the nexus between FQSs and consumers’ choices and preferences [13,14,15]. Several analyses focus on the willingness to pay (WTP), i.e., an estimation of the maximum price a consumer will pay for a food product marketed with a quality scheme, as compared to a conventional one [16,17,18,19]. Moreover, pairing the PDO and PGI labels with other claims has also been studied, taking into consideration consumers’ preferences and WTP for GIs in combination with organic labels [20], although most of the time the studies focused on the trade-offs between PDO/PGI labels and other quality schemes. For instance, de-Magistris and Garcia showed that Spanish consumers are more willing to pay similar price premiums for PDO and organic cheese than for reduced-fat-content cheese [21]. In general, preferences for organic food are related to the idea of naturalness, fairness and environmental impact [22], as well as health awareness and quality [23].

Other studies have analyzed the interest of consumers in GIs combined with the optional quality term Mountain Product. The studies confirmed consumers’ interest and positive attitude towards the Mountain Product label combined with GIs [24], and specifically with the PDO label [25]. Consumers expect mountain farming to be small-scale and mountain products to be healthier than low-land products [26], confirming the growing attention toward active protection of natural resources and a direct involvement in supporting small farmers and local tradition.

Nevertheless, the analysis of the impact of co-branding strategies, e.g., combining PDO label with company brands, as well as organic and/or Mountain Product labels, on consumers’ WTP for agri-food products, is still lacking.

In the present study, we assessed consumers’ preferences for FQSs by means of discrete choice experiments (DCEs) applied to quality cheese products in France and Italy, with the aim of estimating the consumers’ WTP, as well as the grade of trust, use and recognition of multiple labels and quality signs. A DCE simulates consumers’ purchase decisions by studying the influence of attributes (e.g., labels, packaging information, prices, etc.) on consumer preferences [27], and have proven to be predictive of consumers’ market behavior [28].

The experimental study focused on two worldwide-known PDO cheeses: Comté and Parmigiano Reggiano. Comté is a raw-milk, cooked and pressed French PDO cheese produced in three mountain departments located in the Jura Mountains, at the east border with Switzerland (*Official Journal* L 148, 21 June 1996). Comté is the most popular French PDO cheese in terms of volume (64,065 tons in 2015), with a yearly average of 327,000 L of milk produced by each dairy farm [29,30]. Parmigiano Reggiano is probably one of the best-known Italian PDO cheeses in the world (*Official Journal* L 148, 21 June 1996); it is a hard, granular, ripened cheese produced in a diverse territory which includes plain, hills and mountain areas [31,32]. These two products are both managed by a consortium with its own brand, which is perceived by consumers as a quality sign.

Based on the above premises, this paper aimed to test the efficacy of multiple labeling by assessing consumers’ attitudes towards, interest in and willingness to pay for differentiated cheese products. Pairing the PDO scheme with the second label (organic for Comté and Mountain Product for Parmigiano Reggiano) revealed the potential of quality signs that foster production patterns perceived as more sustainable from both an environmental and socio-economic perspective. Indeed, others have indicated, among the collective strategies for increasing the value of GIs, the possibility of adding a higher regulated level of label differentiation between the current PDO and a higher-quality standard (such as the organic or the Mountain Product one; see, e.g., [33]). Adding to the previous research conducted on the same PDO cheeses [34], this work used the stated preferences approach through a hypothetical DCE to estimate consumer demand and WTP. Secondly, it aimed to segment country-specific consumers’ purchase decisions based on the studied cheese attributes. The advantages of a multiple-label system were investigated by combining PDO labels with extra explicit information related to particular production areas, production methods or specific producers/ripening branding strategies, outlining possible strategies for companies.

## 2. Materials and Methods

### 2.1. Theoretical Framework and Model Specification

DCEs stem from the random utility theory (RUT) [35], suggesting that individuals’ utility in a choice situation can be derived from an observable component, defined by the chosen product’s attributes and a random unobservable component [35]:

(1) $$U_{ijt}=\beta_{i}x_{ijt}+\varepsilon_{ijt}$$

where *U_ijt_* is the utility obtained by individual *i* from alternative *j* at the situation *t*; *β_i_* is a vector of part-worth utility, i.e., variables’ parameters for individual *i* estimating his/her preferences; *x_ijt_* is a vector of product attributes; and *ε_ijt_* is the i.i.d. extreme value type 1 stochastic error term. It is assumed that an individual *i* chooses a product alternative *j* (*y_ij_*) if the utility derived from this alternative is maximized compared to the other alternatives:

(2) $$y_{ij}=\{\begin{array}{c} 1,if\;u_{ij}\geq max\left(u_{i}\right)\\ 0\;\;\;\;\;\;\;otherwise \end{array}$$

To estimate the observable component in Equation (1), the standard analytic practice is to pool DCE choice data from individuals and estimate a multinomial logistic regression model [36]. However, assuming that participants have heterogeneous preferences and differ in error variances, other modeling approaches are requested. Among them, the random-parameters logit modeling (RPL) approach extends the traditional multinomial logit models by allowing parameters to randomly vary across individuals, therefore eliciting heterogeneous preferences [37]. Therefore, the unobserved preference heterogeneity among individuals is computed by including a respondent-specific stochastic component specifying the individual deviation from the overall utility mean [37,38]. The probability of individual *i* choosing alternative *j* is [39]:

(3) $$P_{itj}\;\left(\theta \right)=\int^{\;}\frac{\exp\left(\beta_{i}x_{ijt}\right)}{\sum \exp\left(\beta_{i}x_{ijt}\right)}f(\beta_{i}|\theta )d\beta_{i}$$

where *θ* is the vector that specifies the distribution of *β* across sampling participants. In this study, we applied the RPL models on the dummy-coded choice data.

In addition, latent class analysis (LCA) allows us to estimate the individuals’ preferences across different classes. More specifically, the LCA models provide for each individual the probability of each class membership [37], assuming that the overall preference distribution is a combination of unobservable latent segments that are heterogeneous in their utility between the segments and homogeneous within the segment [40,41]. The individuals are assumed to belong to a class s with a certain probability $C_{is}$ for s=1,…,S (where $C_{is}>0$ and $\sum C_{is}=1$; *S* denotes the total number of classes). Thus, the probability of an individual’s membership of segment s will take the following form:

(4) $$C_{is}=\frac{\exp\left(\alpha \lambda_{S}\right)}{\sum_{s=1}^{S}\exp\left(\alpha \lambda_{S}\right)}$$

where *λ_S_* is a vector of the segment-specific parameters, and *α* is the scale factor that is assumed to be equal to one, so each participant has a probability of belonging to a particular segment. For conducting LCA, individual *i*‘s choice probability for alternative *j* in choice situation *t* can then be given as [28,37,39]:

(5) $$P_{ijt}=\overset{S}{\underset{s=1}{\sum }}C_{is}\frac{\exp\left(\beta_{s}x_{ijt}\right)}{\sum_{j=1}^{J}\beta_{s}x_{ijt}}$$

The maximum likelihood approach was used to estimate the LCA model [42]. Besides estimating preferences for different consumer classes, the LCA models also provide the probability of each class membership for each individual [37]. Therefore, due to their properties, we applied the RPL and LCA methods to the country-specific DCE data collected to simultaneously estimate part-worth utility parameters and class membership from the DCE choices.

Furthermore, we computed consumers’ willingness to pay (WTP) for each attribute’s level in each country and segment by dividing the respective attribute level coefficient by the price coefficient [43]:

(6) $$\mathrm{WTP}=\left(\frac{-\beta_{attribute\;level}}{\beta_{price}}\right)$$

Finally, the independent-samples Mann–Whitney U test was applied to test whether country-specific consumer segments significantly differ with respect to the participants’ demographic information, attitude, purchase behavior and food value.

### 2.2. Data Collection and Sample

After receiving the approval from the project coordinating institution, we collected data through a nationwide online survey administered by a third-party company (Lightspeed Research GmbH, Munich, Germany) during summer 2018 to Italian and French adult shoppers. Informed consent was obtained by the company prior to entering the survey. Respondents who were not responsible for their household food shopping were excluded, as well as those who, in the last three months, had not bought cheese [34]. Overall, 1393 participants (N = 747 for Italy and N = 646 for France) joined our online survey originally, of which 687 Italian respondents and 566 French respondents completed the survey, leading to the response rates of 92% for Italian data and 88% for French data. The survey required around 15 min to complete. After data managing and screening out irrelevant samples, our usable valid datasets ended up with the sample size of N = 408 for Italy and N = 400 for France (Table 1); this number far exceeded the sample size estimated by a power analysis assuming three alternatives for each choice task, a 5% margin of error and a desired 95% confidence interval, and is consistent with recommendations for conjoint analysis [44].

There was an equal number of male and female participants in both countries; they were mostly well-educated, with a significantly larger share of higher education in France (university degree or higher). French participants were significantly younger, as the median age was 39 and 44 years in France and Italy, respectively, and were more frequently living in rural areas and in smaller households compared to Italians. Overall, Italian participants were more frequently living in urban areas, and were more likely to have household monthly net incomes of EUR 2500 or less than French ones.

### 2.3. Experimental Design

The online cross-country questionnaire simulating cheese purchase was devised together with academic researchers specialized in the field of agricultural and nutrition science, based on the findings of previous qualitative–quantitative research [45,46] and consisted of three parts. Screening questions were located in the first part of the questionnaire, where participants were asked whether they are (partly) responsible for their household food purchase, and whether they had consumed cheese in the last three months. In the second part of the questionnaire, the choice experiment was carried out, where the cheeses’ attributes and their respective levels were presented graphically in an adequately designed purchasing scenario. The final experimental design of the DCE consisted of three attributes, defined for the cheese alternatives as: quality labels, brands and price (Table 2). These attributes, derived from the previous qualitative analysis [45,46], were proved to be influential in previous studies (see, e.g., [20,21,25]) and are considered to be relatively independent of each other.

**Figure 1:**
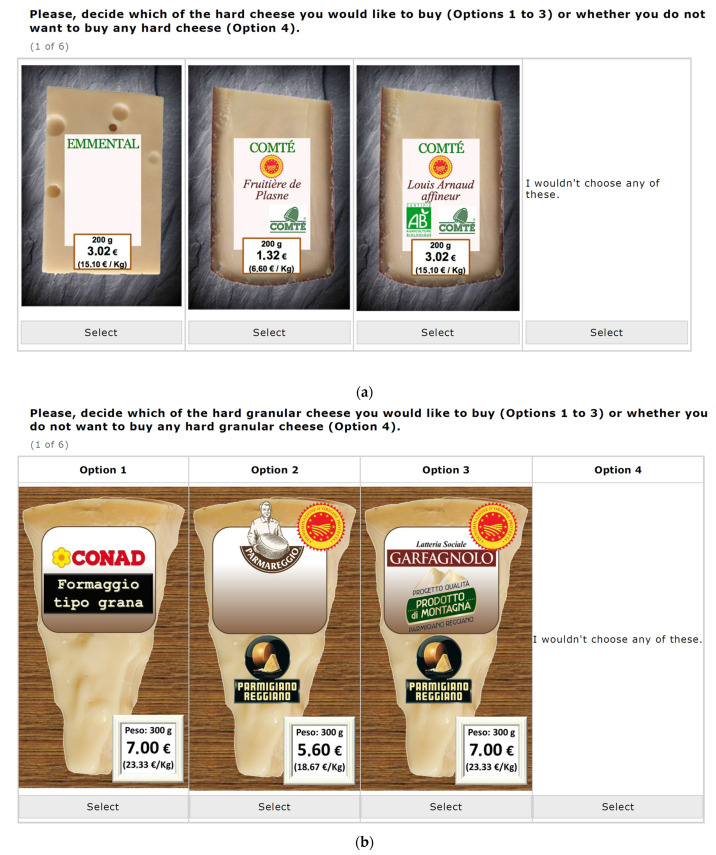
(**a**) An example of choice set in France. (**b**) An example of choice set in Italy.

A cheap talk was used to briefly introduce the participants to the choice experiment, explaining the rationale behind the experiment and the need to respond carefully to the questions. Cheap talk strategies have been proved to eliminate or reduce hypothetical bias [47]. During the DCE, the participants simulated a purchase decision, choosing one of the three presented cheese products, plus an opt-out option. The opt-out or no-choice option gives consumers the alternative of not purchasing the cheese products, which better models real consumers’ purchase contexts. A reduced D-optimal design based on three product alternatives was employed using Ngene software (Version 1.2, ChoiceMetrics, Sydney, NSW, Australia) [48]. Overall, 20 blocking versions of the choice set were generated; participants were requested to answer six randomly assigned choice sets in the survey (Figure 1a,b).

Finally, questionnaire items about participants’ cognitive and affective attitudes towards buying PDO-labeled cheese, perception and purchase habits with respect to cheese shopping, trust in quality labels (i.e., organic, PDO, the Mountain Product), as well as questionnaire items related with the food value questions regarding the importance of motives underlying the food choice were included in the third part of the survey. The questionnaire ended with questions regarding demographic information and socio-economic status. The survey was firstly developed in English, and then translated into French and Italian. Back translations were carefully examined, with minor modifications. Finally, the online questionnaire was pretested prior to the main fielding in order to ensure easy understanding and relevance and that no further changes to the survey were necessary.

## 3. Results

### 3.1. RPL Model Estimates

The solution of the logit analysis was assessed by the likelihood ratio index and by McFadden’s pseudo-R^2^ [49], with the latter being 0.34 and 0.30 in France and Italy, respectively (Table 3). Since values of McFadden’s pseudo-R^2^ from 0.2 to 0.4 indicate an excellent model fit [50], we can conclude that there was a significant impact on consumer choices within the attribute levels presented in the DCE.

The relative importance of attributes for the respondents’ decisions, estimated from the individual perceptions of the cheeses’ attributes importance, indicates that the price had the largest effect on the individuals’ choices, ranging from 52.1% in Italy to 57.5% in France. The next most influential attribute for the respondents was the food quality labels (34.3% and 28.7% in France and Italy, respectively), followed by the brand, which was the least important attribute for both French (8.2%) and Italian consumers (19.2%) (Table 3).

Table 3 shows the results for the country-specific RPL models. The estimated average utility value and standard deviation of each random parameter are reported. The estimated average utilities for the overall model provide evidence of the relative attractiveness of the levels within each attribute. The average utility of the opt-out alternative was computed as the mean value of the individual-specific constants; the negative and significant coefficients for France (−1.34) and Italy (−1.02) indicate that consumers in both countries generally preferred selecting one of the cheese product alternatives in the DCE tasks.

As expected, the RPL results (Table 3) reveal that participants’ preference utility decreased as long as the price of the chosen product increased, in line with the downward sloping demand curve, indicating the negative relationship between the price of a product and the quantity demanded. The results reported in Table 3 also show that participants in both countries, on average, preferred PDO-labeled cheese compared to the unlabeled alternative. Moreover, in both countries, we found a stronger preference for cheese products associated with multiple labels; in France, combining organic and PDO labels for Comté cheese had a greater effect on consumers’ appreciation compared to the no-label product (2.64), more than the PDO label alone (1.13). In Italy, although slightly less evident, this co-labeling preference was also significant; in this case, consumers obtained a higher utility for PDO-labeled Parmigiano Reggiano produced in mountain areas (i.e., with Mountain Product label) compared to the unlabeled alternative (2.01), more than the PDO-labeled option alone (1.46). Not surprisingly, the consumers’ preferences for the proposed brands differed between the two countries because of heterogeneous national contexts. The French model indicates that consumers’ utility was positively affected by choosing the cheese refiner brand and the cheesemaker brand compared to the unbranded option, where the former was slightly more preferred with an average utility of 0.63 than the latter with a utility of 0.43. In Italy, the national dairy company brand provided respondents with a significant increase in individual utility of 1.21 compared to the large-scale retailer brands, whereas the local brand did not provide any significant advantage in terms of respondents’ utility, resulting in a negative utility.

Finally, the resulting standard deviations of the random parameters in the two models were statistically significant, implying a substantial heterogeneity in consumers’ preferences across alternative products. Therefore, a posteriori segmentation based on the choice preference data can reveal differing market opportunities.

### 3.2. Profiling Consumers: Characteristics, Attitudes and Willingness to Pay

A latent class analysis (LCA) was applied to identify the preferences of country-specific consumer segments with different characteristics. Based on the interpretability and comparability of the DCE outputs between France and Italy, the two latent classes’ solution was identified. The resulting *β* coefficients and WTP estimates for each segment in France and Italy are reported in Table 4. Moreover, Table A1 in the Appendix A presents the mean values and standard deviations for several socio-demographic, attitude, purchase behavior and food value variables, as well as the results of the independent-samples Mann–Whitney U test for country-specific consumer groups.

As expected, the coefficients for the price attribute were negative and statistically significant across all classes of the French and Italian samples, suggesting that higher cheese prices generate disutility for consumers (Table 4). In France, the largest class comprised 78% of respondents, which we labeled the *Quality Seekers* class. Members of this class derived the highest utility from the combination of organic and PDO labels for Comté cheese (2.31), or at least the PDO label (1.49), and to a lesser extent the cheese refiner brand (0.44). The farm brand had the lowest part-worth utility in this class (0.28). On average, participants in this class reported a EUR 3.60/200 g marginal WTP for combined organic- and PDO-labeled, instead of unlabeled, purchased cheese products (Table 4). The cheese product with the PDO label alone had, for this segment, a EUR 2.32/200 g WTP compared to the unlabeled alternative.

The marginal WTP estimates for, respectively, the cheese refiner and the farm brands were, on average, 0.69 and EUR 0.43/200 g higher than for the unbranded alternative. The participants in the second class, the *Price-Sensitive, Quality-Adverse* consumers (22% of the total), derived disutility from the two PDO labels. Having a Comté PDO-labeled cheese alone or combined with an organic label reduced consumers’ utility by, respectively, −1.16 and −1.10, compared to having no label (Table 4). For this class, the unlabeled product was preferred over the PDO-labeled one, as the consumers in this class required price discounts for purchasing the Comté PDO and the organic Comté PDO (Table 4). Respondents in this segment reported normally paying a lower price and having generally less positive affective and cognitive attitudes towards buying labeled hard cheese (see purchase behavior and affective and cognitive attitude items reported in Table A1). Regarding perceived barriers, *Price-Sensitive, Quality-Adverse* consumers were more likely to have no time to consider PDO labels when grocery shopping, rarely pay attention to PDO labels while grocery shopping and find it difficult to recognize products with a PDO label in the supermarket (Table A1). *Price-Sensitive, Quality-Adverse* consumers perceived low effectiveness of their behavior; for instance, they believed they are not in a position to have any impact upon farms’ and food processing firms’ behavior. On the other hand, *Quality Seekers* consumers reported generally considered the impact of their purchases on the environment and on other people, and had a stronger belief that each consumer’s behavior can have a positive effect on society by purchasing products produced and sold by companies that behave in a socially and environmentally responsible manner (see perceived effectiveness items in Table A1). This also supports the results shown in Table 4, which presents results for these French consumers and their WTP for the organic product (EUR 3.60/200 g) compared with the discount required by the *Quality-Adverse* shoppers (EUR −3.96/200 g). Trust of *Price-Sensitive, Quality-Adverse* consumers was generally lower than that of *Quality Seekers*; in particular, trust in organic labels and, to a lesser extent, PDO labels, was much lower for respondents in this segment (see trust in labels items reported in Table A1). *Quality Seekers* consumers reported more often checking for the country of origin when buying PDO-labeled products, and were more convinced that, regardless of the country of origin, all PDO-labeled products guarantee the close link between the product and a place or region (Table A1). Finally, for respondents in this segment, it was important that the food eaten on a typical day is healthy and natural (see food choice questionnaire items in Table A1).

In Italy, most of the respondents (89%) were classified into the *High-Quality Seekers* segment (Table 4). Respondents in this class derived the highest utility from the combination of the PDO label for Parmigiano Reggiano cheese and the Mountain Product label (1.16); the PDO label alone had the second highest utility (0.83). Furthermore, the national brand increased the consumers’ utility in this segment compared to the large-scale retailer brand alternative (0.77). On average, participants in this class reported a EUR 2.26/300 g marginal WTP for the cheese product with combined Mountain Product and PDO labeling, instead of no label (Table 4). The cheese product with the PDO label alone had, for this segment, a EUR 1.62/300 g WTP compared to the unlabeled alternative. The marginal WTP estimate for the national brand was, on average, EUR 1.50/300 g higher than the large-scale retailer brand option. The respondents in the second class, the *PDO Lovers* consumers (11% of the total), were less attracted by the combination of the two labels: Mountain Product and PDO labeling (Table 4). They exhibited the highest part-worth utility, compared to the unlabeled alternative, for the Parmigiano Reggiano cheese with the PDO label alone (1.66), whereas the combination of the Mountain Product and PDO labels provided the second highest part-worth utility (1.54). Respondents in this class were also positively influenced by the national brand compared to the large-scale retailer one (1.11). On average, they were willing to pay EUR 0.95/300 g and EUR 0.88/300 g more for, respectively, the cheese product with the PDO label alone, and in combination with the Mountain Product label. Respondents’ WTP for the national brand was EUR 0.63/300 g higher than for the large-scale retailer brand (Table 4). Respondents in the *High-Quality Seekers* segment lived in larger households, and reported normally paying a higher price and purchasing hard cheese more often compared to the *PDO Lovers* consumers (Table A1). Consumers’ affective and cognitive attitude was slightly higher in the *High-Quality Seekers* class, whereas *PDO Lovers* consumers more strongly believed that PDO-labeled hard cheese is too expensive (Table A1). Trust in the EU PDO and Mountain Product labeling system was generally higher in the *High-Quality Seekers* compared to the *PDO Lovers* class. These latter consumers were also less convinced that the PDO-labeled products produced outside of the EU fulfill the same standards as PDO-labeled products produced in the EU. Finally, for respondents in the *High-Quality Seekers* class, it was important that the food eaten on a typical day helps to control their weight (see food choice questionnaire items in Table A1).

## 4. Discussion

Food quality schemes, including PDO, organic and Mountain Product labels, have the strategic advantage of differentiating agricultural and food products on the basis of geographical origin and other specific quality features, such as production methods. However, their effectiveness is subject to the consumers’ perception and WTP. Price premiums in the case of GIs are often product- and GI-specific. Consumers’ WTP for European GI labels is generally positive, with large heterogeneity due to GI labels [2,51]. Moreover, at the international level, PDO cheeses benefit from a price premium and consumers value PDO labels as a quality signal, in particular when GIs are legally protected in the destination country [52]. Other authors have suggested that PDOs are as price elastic as, or more price elastic than, standard products [53]. Our results indicate that price is relatively more important than other attributes in consumers’ selection decisions, indicating that the price attribute had the largest weight in the decision of the individuals. Another recent study conducted in Italy indicated price as the second most important attribute for quality food products, just after hygiene standards [54]. Therefore, as long as prices increase, ceteris paribus, consumers’ utility will decrease more than for other attribute modifications. In particular, the choices of *Price-Sensitive, Quality-Adverse* consumers in France (21.50% of the sample) and *PDO Lovers* in Italy (11.30%) would be particularly affected by price variations. This result indicates that PDO cheese suppliers could not decide on price increases without suffering from reductions in demand, at least from these segments. The high penetration rate of these two PDO-labeled products in the respective mature markets, strongly characterized by strategies such as advertising, social media campaigns and product offerings, may be connected with this relatively high price elasticity.

Several scholars in the past have investigated the effects of GIs, indicating how food with such labels might be perceived to be of higher quality, in particular if produced in a narrow area of origin [55], or if associated with specific quality labels [19]. Our results demonstrate the positive effect of combining multiple labels in France and Italy. It has been noticed that mountain food products represent from 50 to 75% of PDO cheese within the EU [1], whereas the PDO labeling is conceived as an alternative way to protect these products that, in many cases, do not provide the Mountain Product label. Recently, other studies [56] have confirmed the Italian consumers’ interest in the Mountain Product label, relating to the attention paid to environmentally respectful production processes. It has been also suggested that mountain product information might increase the acceptability of local cheese products [57]. Our result demonstrates that the coexistence of the optional term Mountain Product and the PDO label can be effectively promoted by the cheese suppliers, given an adequate control system for preventing frauds. Nevertheless, mountain dairies might implement these strategies to differentiate their PDO cheese from the standard product, owing to higher production costs and price competition. Similarly, the analysis shows that synergies exist between PDO and organic labeling in France. Unlike other studies focusing on the trade-offs between organic and GI labeling [17], our results emphasize the possible synergy between these two certifications. This has also been demonstrated in other food contexts, such extra-virgin olive oil [20], indicating that producers’ marketing efforts might be more effective when quality signals are combined with other quality cues. Similarly, consumers preferring PDO honey often associate this label with environmental sustainability aspects and its organic production [58].

The choice experiment results show heterogeneous consumers’ preferences for the different proposed brands between the two countries due to different national market contexts. In France, the role of the traditional cheesemakers (*fruitière* in French) and cheese refiners (*affineurs*) is recognized as a key factor for obtaining specific sensory characteristics [59]. Here, the chain organization is still largely decentralized, although the penetration of national dairy companies is challenging the sector, and the market reputation of the product is still providing benefits, even to small-scale companies [29]. The French results of the RPL model demonstrate the consumers’ appreciation for the small and medium company brands. In Italy, the national brand is widely recognized in the Italian market and evidently appreciated by the participants; this brand is owned by one of the leading Italian dairy co-operatives, which has had a significant growth in turnover in the past decade, with large investments in marketing strategies [60]. On the other hand, in Italy, the large-scale retailer brand is often associated with the first price option, even when associated with quality-labeled products [61]. The local brand, identifying a small-scale producer brand located in a remote mountain area, is hardly recognized outside the restricted local provenance.

Other researchers have indicated France and Italy as countries clearly PDO/PGI-oriented, reporting high consumer awareness of geographical indications, especially PDO, a strong tradition of using this quality scheme and higher interest in obtaining information through quality labels [11]. Similarly, different studies have reported a high level of awareness in Southern European countries, such as France and Italy, compared to Northern European ones [62]. This justifies the larger size of the *Quality Seekers* segment in the French sample, and the general PDO-labeled cheese appreciation across the two Italian classes. Moreover, Italy is the country with the highest number of geographical designations recognized by the European law among food products in general, as well as more specifically in the case of cheese products [1]. These two large segments with strong awareness and appreciation for PDO labels have relevant implications for the cheese industry. Consumers’ ability to recognize and use these labels has important effects for companies’ competitiveness, even in international markets. The foreign demand increases as long as consumers recognize the PDO label as a quality signal. Recent empirical analysis conducted in the French cheese industry shows that this international demand effect is higher than the increase in production costs, due to high-quality ingredients or additional production tasks, and that PDO labels have a role in firm export competitiveness [52].

In the literature, the effects of socio-demographic characteristics on consumers’ behavior towards mountain labels are conflicting. Some studies found that consumers most likely to be interested in mountain food products are older, and more often women [63], whereas others have indicated that young people choose more frequently the product with the mountain logo [56]. In the present study, the socio-demographic variables had only a marginal effect in defining the market segments. In this regard, the socio-demographics differences across the French and Italian samples, in terms of age, living location, education level, income and household size, did not allow us to compare different clusters across countries. For instance, the average difference of 5 years between the *Price-Sensitive, Quality-Adverse* cluster in France (38 years) and the *PDO Lovers* consumers in Italy (43 years) is due also to the overall age difference between the two samples.

Nevertheless, the attitude variables were more relevant in shaping the segments’ characteristics. For instance, affective and cognitive attitude were more relevant in the *Quality Seekers* cluster, whereas perceived barriers were more important for *Price-Sensitive*, *Quality-Adverse* consumers in France. These results are in line with theoretical frames investigating the role of attitude and beliefs in affecting individuals’ behavior (see, e.g., the Theory of Planned Behavior [64]). The perceived effectiveness was also significantly different in the two French classes, indicating that those aware of the environmental and social consequences of individuals’ purchasing decisions are more frequently part of the *Quality Seekers* cluster. A similar path was also found in Italy for the *High-Quality Seekers* segment. This result is similar to what was found by Mazzocchi and colleagues [65], showing that the attitude in believing and acting as “green consumers” had a positive effect on the choice of cheese with the mountain label. The significantly higher trust in the EU PDO and Mountain Product labeling system of *High-Quality Seekers* class in Italy is in line with previous findings showing that consumers more interested in mountain products are more likely to appreciate stricter rules by the EU government on the mountain origin of raw materials and on processing in mountain areas [63]. Finally, other studies have shown that trust in the EU PDO labelling system is positively correlated with the intention to purchase and the purchase frequency of PDO cheese in Italy [13,34,66].

Although we do not claim the results of our study cover the whole complex cheese market, they could help practitioners and stakeholders in the dairy supply chain to better understand consumer motivations, providing them with concrete tools to more strongly promote and communicate particular production choices in order to foster a competitive advantage. A multiple-label system could be adopted by producers to stress their specific production processes or raw material selection, and to communicate with consumers in an effective and transparent way. These mountain and organic labeling systems, combined with the PDO labels, give dairy companies more opportunities for promoting sustainable practices, achieving a competitive advantage over standard competitors, and affecting consumers’ willingness to pay a price premium for such products. Moreover, by segmenting the studied market based on the estimated consumers’ WTP, this research has identified relevant groups of consumers with similar preferences and attitudes.

The main limitation of this study is that the observations are based on a hypothetical experiment, which did not imply an actual purchase decision by respondents. However, the introduction of a cheap talk at the beginning of the experiment should have minimized the hypothetical bias [47]. Secondly, in order to approach the representativeness of the Italian and French populations, the quota sampling procedure based on the national census statistics with respect to gender and age was applied during our recruitment process in this study. Focusing on income and educational level might have led to more accurate representativeness of the sample. Then, the application of two different label combinations, i.e., the PDO label with, respectively, the organic label in France and the Mountain Product label in Italy, might have reduced the cross-country comparability. However, the specific marketing contexts were carefully considered in this choice. For instance, a specification for the Mountain Product label already exists in the Parmigiano Reggiano district [67], whereas this does not apply in the French case. Moreover, the Parmigiano Reggiano consumers’ perception of eco-friendly attributes has been recently studied [68]. For these reasons, two labeling systems were combined with the PDO one, providing wider evidence of consumers’ preferences across different quality labels, while partially sacrificing cross-country comparability. Finally, it is also possible that other dimensions, such as participants’ prior knowledge of cheese and food fraud, not investigated in the present study may have affected their choices and WTP [69]. Despite these limitations, we contribute to (1) understating consumer WTP for cheese products with different quality labeling, (2) examining how consumers’ WTP varies across segments with different attitudes and characteristics and (3) exploring synergies across multiple-label cheese products.

## 5. Conclusions

In summary, the present study provided evidence of the consumers’ WTP for differentiated Comté and Parmigiano Reggiano PDO cheeses in France and Italy. The random-parameter Logit estimates, applied to data collected with a discrete choice experiment in two samples of Italian and French consumers, showed that price was the most important attribute. This was followed by the PDO quality label, particularly when paired with other quality features (i.e., the organic label in France, and the Mountain Product label in Italy). In addition, latent class analysis indicated two segments in the French and Italian samples, presenting heterogeneous attitudes towards quality-labeled food products and personal characteristics.

## Figures and Tables

**Table 1:** Sample demographic and socio-economic characteristics, number (*n*), percentage (%), mean and standard deviations (SD), median and interquartile range (IR).

Socio-Demographic Categories		France		Italy		All		*p*-Value
		*n* = 400		*n* = 408		*n* = 808		
		*n*	%	*n*	%	*n*	%	
Food purchase responsibility	Responsible	288	72.0	260	63.7	548	67.8	0.012 ^a^
	Co-responsible	112	28.0	148	36.3	260	32.2	
Gender	Female	200	50.0	203	49.8	403	49.9	0.944 ^a^
	Male	200	50.0	205	50.2	405	50.1	
Age (years)	Mean (SD)	40.0 (14.0)		42.9 (12.6)		41.5 (13.3)		0.003 ^b^
	Median (IR)	39.0 (27.0–51.0)		44.0 (34.0–54.0)		42.0 (31.0–53.0)		
Living area	Rural area	198	49.5	52	12.7	250	30.9	<0.001 ^a^
	Urban medium town	100	25.0	171	41.9	271	33.5	
	City	102	25.5	185	45.3	287	35.5	
Education	Lower secondary/primary education or lower	18	4.5	29	7.1	47	5.8	<0.001 ^a^
	Upper secondary education	127	31.8	157	38.5	284	35.1	
	University or college entrance qual.	110	27.5	67	16.4	177	21.9	
	Bachelor’s degree or equivalent level	82	20.5	67	16.4	149	18.4	
	Master, postgraduate or doctoral degree	63	15.8	88	21.6	151	18.7	
Household monthly net income	(FR) < EUR 1130/(IT) < EUR 900	46	11.5	29	7.1	75	9.3	<0.001 ^a^
	(FR) EUR 1131–EUR 1450/(IT) EUR 901–EUR 1500	26	6.5	75	18.4	101	12.5	
	(FR) EUR 1451–EUR 2090/(IT) EUR 1501–EUR 2500	83	20.8	126	30.9	209	25.9	
	(FR) EUR 2091–EUR 2890/(IT) EUR 2501–EUR 3500	74	18.5	88	21.6	162	20.0	
	(FR) EUR 2891–EUR 4100/(IT) EUR 3501–EUR 4500	98	24.5	24	5.9	122	15.1	
	(FR) ≥ EUR 4101/(IT) ≥ EUR 4501	50	12.5	7	1.7	57	7.1	
	Prefer not to answer	23	5.8	59	14.5	82	10.1	
Household size ^1^	Mean (SD)	2.6 (1.2)		3.1 (1.1)		2.9 (1.2)		<0.001 ^b^
	Median (IR)	2.0 (1.0–3.0)		3.0 (2.0–4.0)		3.0 (2.0–4.0)		
Number of children ^2^	Mean (SD)	0.6 (0.9)		0.5 (0.8)		0.6 (0.9)		0.169 ^b^
	Median (IR)	0.0 (0.0–1.0)		0.0 (0.0–1.0)		0.0 (0.0–1.0)		

^1^ Number of persons in household. ^2^ <18-year-old persons in a household. ^a^ Pearson chi-square. ^b^ Mann–Whitney *U* Test.

**Table 2:** Attributes and levels used in the DCE in France and Italy.

Attribute/Levels	France	Italy
Food quality labels	No-label semi-hard cheese	No-label hard granular cheese
	Comté PDO 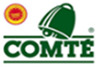	Parmigiano Reggiano PDO 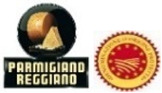
	Organic + Comté PDO 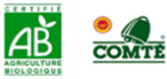	Mountain Product + Parmigiano Reggiano PDO 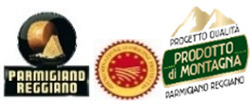
Brand	No brand	Large-scale retailer brand 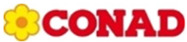
	Farm brand (i.e., Fruitière de Plasne)	National brand 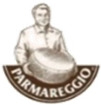
	Cheese refiner brand (i.e., Louis Arnaud affineur)	Local brand 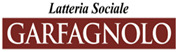
Price	Level 1: EUR 1.32/200 g	Level 1: EUR 5.60/300 g
	Level 2: EUR 2.17/200 g	Level 2: EUR 6.30/300 g
	Level 3: EUR 3.02/200 g	Level 3: EUR 7.00/300 g
	Level 4: EUR 3.87/200 g	Level 4: EUR 7.70/300 g

**Table 3:** Random-parameter logit model for DCE data.

France (*n* = 400)			Italy (*n* = 408)		
Attribute/Levels	Relative Importance (%)	Average Utilities (SD)	Attribute/Levels	Relative Importance (%)	Average Utilities (SD)
Food quality labels	34.33		Food quality labels	28.71	
Comté PDO vs. No label		1.13 (2.06) ***	Parmigiano Reggiano PDO vs. No label		1.46 (1.32) ***
Organic + Comté PDO vs. No label		2.64 (3.30) ***	Mountain Product + Parmigiano Reggiano PDO vs. No label		2.01 (1.79) ***
Brands	8.20		Brands	19.15	
Farm brand vs. No brand		0.43 (1.05) ***	National brand vs. Large-scale retailer brand		1.21 (2.05) ***
Cheese refiner brand vs. No brand		0.63 (1.18) ***	Local brand vs. Large-scale retailer brand		−0.16 (1.29)
Price	57.47		Price	52.14	
Level 2 vs. Level 1		−0.64 (1.40) ***	Level 2 vs. Level 1		−0.78 (1.08) ***
Level 3 vs. Level 1		−2.21 (2.59) ***	Level 3 vs. Level 1		−2.14 (1.79) ***
Level 4 vs. Level 1		−4.40 (3.60) ***	Level 4 vs. Level 1		−3.65 (2.69) ***
Constant (opt-out option)		−1.34 ***	Constant (opt-out option)		−1.02 ***
Wald chi-square	357.17		Wald chi-square	456.23	
Prob > chi-square	0.00		Prob > chi-square	0.00	
Pseudo R-square	0.34		Pseudo-R-square	0.30	
Null loglikelihood	−3327.11		Null loglikelihood	−3393.65	
Restricted loglikelihood	−2196.09		Restricted loglikelihood	−2381.56	
Likelihood ratio test: prob > chi-square	0.000		Likelihood ratio test: prob > chi-square	0.000	

Sig: *** *p* < 0.001.

**Table 4:** Latent class analysis (LCA) models: β -coefficient estimates and willingness to pay (WTP) estimates (expressed in EUR /200 g for France, EUR /300 g in Italy).

	France (*n* = 400)					Italy (*n* = 408)			
	Price-Sensitive, Quality-Adverse		Quality Seekers			High-Quality Seekers		PDO Lovers	
	21.50%		78.50%			88.70%		11.30%	
Attribute/Level	*β*	WTP	*β*	WTP	Attribute/Level	*β*	WTP	*β*	WTP
Opt-out option	−0.909 ***		−2.834 ***		Opt-out option	−3.171 ***		0.146	
Comté PDO vs. No label	−1.156 ***	−4.16 ***	1.489 ***	2.32 ***	Parmigiano Reggiano PDO vs. No label	0.829 ***	1.62 ***	1.663 ***	0.95 ***
Organic + Comté PDO vs. No label	−1.101 ***	−3.96 ***	2.307 ***	3.60 ***	Mountain Product + Parmigiano Reggiano PDO vs. No label	1.157 ***	2.26 ***	1.537 ***	0.88 ***
Farm brand vs. No brand	0.138	n.s.	0.277 **	0.43 **	National brand vs. Large-scale retailer brand	0.767 ***	1.50 ***	1.113 ***	0.63 ***
Cheese refiner brand vs. No brand	0.110	n.s.	0.444 ***	0.69 ***	Local brand vs. Large-scale retailer brand	0.012	n.s.	−0.192	n.s.
Price	−0.278 ***		−0.641 ***		Price	−0.512 ***		−1.755 ***	

Sig: ** *p* < 0.01; *** *p* < 0.001. n.s.: not significant.

## Data Availability

The data are available on request from the corresponding author.

## Appendix A

**Table A1:** Latent class analysis (LCA) models: participants’ characteristics, attitude, purchase behavior differences between classes in France and Italy (independent-samples Mann–Whitney U test).

Variables	France (*n* = 400)					Italy (*n* = 408)				
	Price-Sensitive, Quality-Adverse		Quality Seekers			High-Quality Seekers		PDO Lovers		
	21.50%		78.50%			88.70%		11.30%		
	Mean	Sd	Mean	Sd	*p*-Value	Mean	Sd	Mean	Sd	*p*-Value
*Socio-demographics*										
Gender	1.53	0.50	1.49	0.50	0.542	1.51	0.50	1.43	0.50	0.330
Age	38.27	15.29	40.52	13.58	0.157	42.85	12.46	43.13	13.48	0.859
Living Location	1.69	0.79	1.78	0.85	0.483	2.32	0.70	2.37	0.57	0.884
Education	2.95	1.15	3.16	1.15	0.183	3.10	1.31	2.83	1.25	0.207
Income	3.73	1.80	4.05	1.62	0.096	3.67	1.71	3.35	1.84	0.141
Household Size	2.49	1.35	2.62	1.24	0.295	3.14	1.08	2.78	1.19	0.042
*Purchase behavior*										
What price do you normally pay for one package of 200 g hard cheese?	3.91	2.23	4.26	1.84	0.030	3.24	2.00	2.11	1.68	0.000
On average, how often do you buy hard cheese?	3.20	1.65	3.30	1.47	0.329	3.28	1.36	2.76	1.18	0.019
On average, how often do you eat hard cheese?	3.33	1.72	3.50	1.48	0.182	3.94	1.34	3.83	1.37	0.490
*Affective attitude*										
Buying PDO labeled hard cheese instead of hard cheese without such a label would make me feel...										
Unsatisfied/ Satisfied	5.13	1.20	5.65	1.26	0.000	5.43	1.50	5.00	1.41	0.011
Unhappy/Happy	5.02	1.28	5.24	1.61	0.025	5.10	1.71	4.70	1.41	0.022
Bad/Good	5.12	1.34	5.38	1.54	0.017	5.20	1.68	4.89	1.43	0.062
*Cognitive attitude*										
I think that buying PDO labeled hard cheese instead of hard cheese without such a label is…										
Meaningless/Meaningful	4.92	1.10	5.42	1.22	0.000	5.46	1.41	5.11	1.20	0.020
Harmful/Beneficial	5.11	1.42	5.43	1.53	0.015	5.31	1.55	5.02	1.27	0.037
Unimportant/Important	4.72	1.55	5.38	1.43	0.000	5.54	1.46	5.02	1.26	0.002
*Perceived barriers*										
PDO labeled hard cheese is too expensive	4.60	1.44	4.74	1.29	0.464	4.40	1.49	4.80	1.85	0.049
I rarely pay attention to PDO labels while grocery shopping	4.44	1.66	3.80	1.79	0.006	3.38	1.83	3.41	1.38	0.755
There is no PDO labeled hard cheese of my preferred brand available in the store where I generally do my grocery shopping	3.66	1.49	3.35	1.63	0.089	3.23	1.86	3.24	1.43	0.919
I have no time to consider PDO labels when grocery shopping	4.09	1.53	3.27	1.70	0.000	3.26	1.84	3.07	1.25	0.732
I find it difficult to recognize products with a PDO label in the supermarket	4.01	1.55	3.53	1.64	0.016	3.25	1.81	2.93	1.55	0.328
*Perceived effectiveness*										
When I buy products, I consider the impact my purchase has on the environment and on other people	4.33	1.44	4.83	1.35	0.002	4.90	1.39	4.46	1.28	0.011
Since one single person cannot have any impact upon how farms and food processing firms behave, it does not make any difference what I do	4.15	1.57	3.47	1.78	0.001	3.64	1.75	3.24	1.64	0.144
Each consumer’s behavior can have a positive effect on society by purchasing products produced and sold by companies that behave in a socially and environmentally responsible manner	4.96	1.38	5.50	1.18	0.001	5.43	1.30	5.26	1.31	0.356
*Trust in labels*										
Products with the EU PDO label fulfil strict rules	4.98	1.25	5.27	1.15	0.023	5.39	1.25	4.93	1.44	0.018
The EU PDO label guarantees that the products are of a higher quality	4.92	1.25	5.13	1.24	0.097	5.40	1.31	4.93	1.29	0.010
I have great trust in the control system behind the EU PDO label	4.75	1.32	5.06	1.25	0.033	5.24	1.39	4.83	1.40	0.040
Products with the organic label fulfil strict rules	4.94	1.37	5.43	1.22	0.001	4.94	1.27	4.46	1.15	0.011
The organic label guarantees that the products are of a higher quality	4.67	1.45	5.19	1.41	0.001	5.17	1.29	4.70	1.35	0.013
I have great trust in the control system behind the organic label	4.67	1.50	5.26	1.38	0.000	5.04	1.28	4.28	1.22	0.000
*Equality label standards*										
PDO labeled products produced outside of the European Union fulfil the same standards as PDO labeled products produced in the European Union	3.98	1.42	4.06	1.63	0.565	3.89	1.74	3.28	1.39	0.020
PDO labeled products from other countries of the European Union fulfil the same standards as PDO labeled products produced in France	4.15	1.40	4.32	1.45	0.337	4.27	1.55	3.70	1.63	0.024
I check the country of origin when I buy PDO labeled products	4.56	1.55	5.16	1.42	0.001	5.31	1.42	5.02	1.64	0.265
I am convinced that, regardless of the country of origin, all PDO labeled products guarantee the close link between the product and a place or region	4.69	1.21	5.00	1.23	0.018	4.68	1.48	4.11	1.59	0.017
*Food choice questionnaire*										
It is important to me that the food I eat on a typical day…										
is healthy	5.29	1.33	5.84	1.05	0.001	5.07	1.18	4.72	1.50	0.277
is a way of managing my mood (e.g., a good feeling or coping with stress)	4.45	1.44	4.64	1.40	0.187	5.16	1.21	5.00	1.32	0.565
is convenient (in buying and cooking)	5.06	1.24	5.15	1.08	0.420	5.79	1.02	5.72	1.28	0.898
provides me with pleasure (e.g., appearance, texture, smell, taste)	5.53	1.27	5.84	1.06	0.057	5.70	1.14	5.41	1.39	0.247
is natural (no additives, only natural ingredients)	5.13	1.32	5.71	1.14	0.000	4.91	1.36	4.98	1.44	0.643
is affordable	5.38	1.26	5.56	1.09	0.296	4.98	1.37	4.72	1.44	0.207
helps me control my weight	4.48	1.43	4.72	1.42	0.115	4.79	1.44	3.87	1.65	0.000
is familiar	4.95	1.18	5.02	1.10	0.495	5.37	1.30	5.20	1.29	0.323
is environmentally friendly	5.12	1.35	5.40	1.20	0.056	5.43	1.29	5.15	1.56	0.314
is animal friendly	5.14	1.27	5.39	1.29	0.059	5.20	1.19	5.11	1.34	0.605
is produced and traded in a fair manner	5.05	1.30	5.26	1.20	0.107	5.79	1.10	5.57	1.26	0.263
